# *CD24* and *APC* Genetic Polymorphisms in Pancreatic Cancers as Potential Biomarkers for Clinical Outcome

**DOI:** 10.1371/journal.pone.0134469

**Published:** 2015-09-22

**Authors:** Sivan Shamai, Ilana Nabiochtchikov, Sarah Kraus, Sally Zigdon, Dina Kazanov, Michal Itzhak-Klutch, Carmit Eizner, Nadir Arber, Ravit Geva

**Affiliations:** 1 Department of Oncology, Tel Aviv Sourasky Medical Center, Tel Aviv, Israel; 2 The Integrated Cancer Prevention Center, Tel Aviv Sourasky Medical Center, Tel Aviv, Israel; 3 Sackler School of Medicine, Tel Aviv University, Tel Aviv, Israel; Thomas Jefferson University, UNITED STATES

## Abstract

**Background:**

There are no validated biomarkers that correlate with the prognosis of pancreatic ductal adenocarcinoma (PDA). The *CD24* and adenomatous polyposis coli (*APC*) genes are important in the malignant transformation of gastrointestinal cells. This study examined *APC* and *CD24* genetic polymorphisms and their possible impact on survival of patients with PDA.

**Methods:**

Clinical and pathological data as well as blood samples for extracting DNA were obtained for 73 patients with PDA. Real-time PCR assessed genetic variants of *APC* (I1307K and E1317Q), and four different single nucleotide polymorphisms (SNPs) in the *CD24* gene: C170T (rs52812045), TG1527del (rs3838646), A1626G (rs1058881) and A1056G (rs1058818).

**Results:**

The median age at diagnosis was 64 (41–90) years. Thirty-one patients (42.5%) were operable, 16 (22%) had locally advanced disease and 26 (35.5%) had disseminated metastatic cancer. The malignancy-related mortality rate was 84%. Median survival was 14 months (11.25–16.74). Survival was similar for wild-type (WT), heterozygous and homozygous variants of the *APC* or *CD24* genes. The three most frequent *CD24* SNP combinations were: heterozygote for A1626G and WT for the rest of the alleles (14% of patients), heterozygote for C170T, A1626G, A1056G and WT for the rest (14% of patients), and heterozygote for C170T, A1056G and WT for the rest (10% of patients). All patients were APC WT. The first two groups were significantly younger at diagnosis than the third group.

**Conclusions:**

Specific polymorphisms in the *APC* and *CD24* genes may play a role in pancreatic cancer development. Correlation with survival requires a larger cohort.

## Introduction

Pancreatic ductal adenocarcinoma (PDA) is known for its high mortality and dismal prognosis. The overall 5-year survival rate is <6%. In the US in 2015, an estimated 48,960 new patients will be diagnosed, and 40,560 patients will die from their illness.[1]. PDA is caused as a result of mutations in cancer-associated genes, the majority of which are sporadic, and only about 15% are germline mutations. Sporadic mutations affect several key genes. An early-onset mutation appears in the *K-Ras* oncogene in 90% of the cases. Other typically affected genes are tumor suppressor genes, such as *TP53*, *CDKN2A* and *SMAD4*, with new candidate drivers of pancreatic carcinogenesis recently identified (*KDM6A* and *PREX2*) [2]. Mutations in moderately penetrant susceptibility genes, such as *BRCA2*, *CDKN2A*, and *MLH1*, account for <5% of pancreatic cancers, suggesting that much of the inherited risk to this disease may be due to low-penetrance common genetic variants [3]. Chemotherapy has little success in recurrent or disseminated pancreatic cancer. The best therapeutic results have been achieved with a combination of three different chemotherapy agents, yielding a median survival of less than one year [4]. These figures highlight the need for further research and understanding of the molecular pathways driving this malignancy.

CD24 is a membrane mucin-like protein, anchored by glycosylphosphatidylinositol (GPI), and its core protein is composed of 27–31 amino acids. It is expressed predominantly on hematopoietic cells, mostly B-cell precursors, cells of the central nervous system and epithelial cells [5–7]. It functions as an adhesion molecule, binding to P-selectin on platelets and endothelial cells, as well as to L1 protein expressed on lymphoid and neural cells [5]. The *CD24* gene has several genetic variants, arising from single nucleotide polymorphisms (SNPs). These include 170^C→T^, a polymorphism that leads to an amino acid substitution from Alanine to Valine, in a location connected to membrane linking through the GPI anchor [8–9]. Three additional SNPs are located in the 3'–untranslated region, and they include 1626^A→G^, 1056^A→G^ and 1527^tgde^, which may affect *CD24* mRNA stability.

Previous studies have shown that CD24 is a marker for a variety of cancer stem cells, including pancreatic cancer [10]. Changes in CD24 expression in cancer cell lines can alter cellular properties and tumor growth. We had previously shown that treatment of pancreatic and colon cancer cell lines with anti-CD24 monoclonal antibodies (MAbs) or CD24 downregulation using short hairpin (sh)RNA effectively inhibited cell proliferation *in vitro* and retarded tumorigenicity in xenografted nude mice [11]. The role of *CD24* in PDA is still unclear. It has been linked to poor differentiation, but not to survival [12]

The tumor suppressor gene, *APC* (adenomatous polyposis coli), has been extensively investigated and linked to colorectal cancer development [13,14]. Laken et al. reported a germline missense mutation that caused the substitution of T to A at nucleotide 3977, leading to the insertion of lysine (K) instead of isoleucine (I) at codon 1307 (I1307K). This is believed to cause instability, thus possibly contributing to malignant transformation [15]. Another polymorphism, the *APC* E1317Q variant, is a substitution of glutamic acid (E) for glutamine (Q) at codon 1317 (E1317Q). This results from a G to C substitution at nucleotide 4006 and may be linked to cancer development [16].

The role of *APC* in PDA is not clear. It has been shown that pancreatic tumors failed to develop following conditional inactivation of *APC* in the pancreas, suggesting that *APC* is required for tumorigenesis in the pancreas [17]. An earlier study that examined *APC* E1317Q and I1307 in PDA found no association in a cohort of 58 patients [16]. The current study aimed to investigate a possible association between the clinical course of PDA and genetic alterations in the *CD24* and *APC* genes.

## Materials and Methods

### Participants

Newly diagnosed patients with PDA who were treated at the Department of Oncology at Tel Aviv Sourasky Medical Center, Tel Aviv, Israel, between 2000–2014, were prospectively recruited to the study. After obtaining written informed consent, blood samples were taken for analysis and genotyping of the *CD24* and *APC* SNPs. The patient's files were retrospectively viewed, and clinical and pathological data were collected, including demographics, disease stage, treatment, response to chemotherapy and survival. The trial was approved by the local Institutional Review Board (IRB, Sourasky medical center Helsinky committee) and the Israeli Ministry of Health (Helsinky approval number 02–130, Israeli ministry of health application 919990171).

### Genetic Analysis

#### Assay methods

Blood was collected in tubes containing EDTA. Peripheral blood leukocytes (PBLs) were isolated from whole blood samples by collecting white buffy coats obtained after blood centrifugation for 3 minutes at 4°C at 3,000 rpm and discarding the plasma supernatant.

### DNA Extraction

Genomic DNA was extracted from PBLs by standard methods as described by Miller et al. [18].

### Determination of the Different *CD24* Polymorphisms

Real-time PCR (qPCR) was used to genotype rs8734 (C170T), rs3838646 (TG1527del), rs1058881 (A1626G), and rs1058818 (A1056G) *CD24* polymorphisms using Custom TaqMan SNP Genotyping Assays predesigned by Applied Biosystems (Applied Biosystems, Foster City, CA, USA), following the technical procedures recommended by the manufacturer. The assay reagents for SNP genotyping from the Assays-by-design consisted of a 40X mix of unlabeled PCR primers and TaqMan MGB probes (FAM and VIC dye-labeled). These assays were designed for the genotyping of the specific SNPs. The following primer sequences were used:

For the rs8734 polymorphism: forward primer, GGTTGGCCCCAAATCCA; reverse primer, GACCACGAAGAGACTGGCTGTT; allele 1 (VIC), CACCAAGGCGGCT; allele 2 (FAM), CACCAAGGTGGCTG.

For the rs1058818 polymorphism: forward primer, AGCTAAACGGATTCCAAAGAGTAGAA; reverse primer, TGGGCGACAAAGTGAGACTGT; allele 1 (VIC), TGCATTGACCACGACT; allele 2 (FAM), TGCATTGACCGCGAC.

For the rs3838646 and rs1058881 polymorphisms: Assay ID AH0JB89 and Assay ID AH1SAFH, respectively (Applied Biosystems).

The conditions for PCR amplification were identical. The PCR reactions were carried out in a 10 μl volume containing 20 ng of genomic DNA and prepared using TaqMan Universal PCR Master Mix components (Applied Biosystems), which contained nucleotides, buffer, uracil-N-glycosylase, amplitaq, and a passive reference dye (ROX). The reaction mixture contained 5.0μl of TaqMan Universal PCR Master Mix, 2.75μl of H_2_O, 0.25μl of assay components (primer set and probe specific for each polymorphism), and 2.0μl of DNA from each sample. A Step One Plus Real-Time PCR System (Applied Biosystems) was used to perform the qPCR experiments using the following cycling protocol. The cycling was initiated by pre-heating at 600°C for 30 seconds and 950°C for 10 minutes, followed by 40 cycles of 920°C for 15 seconds, 600°C for 1 minute and 600°C for 30 seconds. The Stepone v2.2 (Applied Biosystems) program was used to interpret the reaction results, using the graphical representation of the VIC and FAM fluorophore emissions with respect to constitutive ROX emissions.

### Assessing the I1307K Polymorphism at the *APC* Gene

Genomic DNA was amplified by PCR using a forward primer (5' –GAAATAGGATGTAATCAGACG – 3') and a reverse primer (5' –AGTCTGCTGGATTTGGTTCTA – 3'). For real time-PCR, a sensor primer was designed according to the wild-type (WT) allele and downstream to it as an anchor primer. For the detection of the specific polymorphic nucleotide (T/A at position 3977 of SEQ ID NO: 7), the anchor primer was: LC-Red 640- TTTGCAGGGTATTAGCAGAATCTGCTTCCTGTG–ph (SEQ ID NO: 9) and the sensor primer was: CCAATCTTTTCTTTTTTTTCTGC–FL (SEQ ID NO: 10).

### Assessing the E1317Q Polymorphism at the *APC* Gene

Genomic DNA was amplified by PCR using a forward primer (5'–GAAATAGGATGTAATCAGACG– 3') and a reverse primer (5'–CACCACTTTTGGAGGGAGA– 3'). Primers and detection of the specific polymorphic nucleotide (G/C at position 4006 of SEQ ID NO: 7) was by real-time PCR using the anchor primer: TGCTGTGACACTGCTGGAACTTCGC-FL (SEQ ID NO: 11) and sensor primer (ph-LC-Red705-CACAGGATCTTGAGCTGACCTAG (SEQ ID NO: 12).

### Statistical Analysis

Descriptive statistical values for all variables were calculated for the entire study population. These included the median, range, frequencies and relative frequencies as applicable according to variable type. The relative frequencies of each gene permutation (combination) were calculated, and the three most frequent permutations were further analyzed. Descriptive statistics were calculated for each permutation group. Differences in categorical variables between the various permutation groups were analyzed using the Chi-squared test of independence. Differences in demographic quantitative variables were analyzed by nonparametric ANOVA with the Kruskal-Wallis test (for omnibus tests) and further with the Wilcoxon rank-sum test if pair comparisons were indicated by the former. Differences in survival curves were analyzed using the Log-rank test. A *p*-value <0.05 was considered statistically significant. When applicable, tests were two-sided and adjusted for multiple comparisons. All statistical analyses were conducted using SPSS 22 (IBM Corporation Inc., USA).

A power analysis for Log Rank test was based ona 2-sided alpha level of 0.05. The genotypes ratio was based on previous studies, (19,20). Wt group was taken as reference, with a supposed survival of 12 months for the studied population (treated pancreatic cancer patients) and compared to non-wt genotypes. Power for symmetrical difference in survival (i.e. wt equally likely to be better or worse than comparison group) was calculated, given the exploratory nature of this study, with a clinically significant difference defined as 4 months (S1 Data)

## Results

### Clinical Characteristics of the Patients

The records of 73 PDA patients who had been treated at our institution between 2000 to 2014 were available for both genetic and clinical assessments (Table 1). They included 38 (52%) males and 35 (48%) females with a median age of 64 years (41–90). Ten patients (14%) had previous malignancy with no recurring pattern. At diagnosis, 31 (42.5%) patients were operable, 16 (21.9%) had locally advanced inoperable disease, and 26 (35.6%) had metastatic cancer. Thirty-four patients underwent surgery with curative intent: 22 had the Whipple procedure, nine had a distal pancreatectomy and three had a total pancreatectomy. Twenty-six patients (76.5%) had R0 resection and eight patients (23.5%) had R1 resection. Of the thirty-four patients who were operated, seventeen (50%) had no lymph node involvement, while seventeen (50%) had pathological lymph nodes [median: 1, range (1–13)]. Thirteen of the 16 patients (81%) who had locally advanced inoperable disease received neoadjuvant chemotherapy, most commonly gemcitabine and cisplatin, and nine received radiotherapy, with concurrent weekly cisplatin. At the time of last appraisal, 13 of the 73-patient cohort (17.8%) remained free of disease, 56 (76.7%) had disseminated disease, and 4 (5.5%) were lost to follow-up.

**Table 1:** Clinical Characteristics of All Patients (N = 73).

Characteristic	N (%)
**Gender**	
Male	38 (52%)
Female	35 (48%)
**Median age at diagnosis**	64 (41–90)
	
**Stage at diagnosis**	
Localized	31 (42.5%)
Locally advanced	16 (21.9%)
Metastatic	26 (35.6%)
	
**Localized treatment**	
Operated with curative intent	31 (100%)
Adjuvant chemotherapy	17 (55%)
	
**Locally advanced treatment**	
Neoadjuvant chemotherapy	13 (81%)
Neoadjuvant chemo-radiotherapy	9 (56%)
Surgery	3 (18.7%)
	
**Diagnosed as metastatic at any time**	
Yes	56 (77%)
No	13 (18%)
Missing data	4 (5%)
	
**Treatment for metastatic disease**	
Best supportive care	16 (28%)
One treatment line	23 (41%)
Two and more treatment lines	17 (30%)

The patients who had metastatic disease received a median of one treatment line (range 0–5). The most common first treatment option was gemcitabine-based doublet (16/56, 28%), a clinical trial (14/56, 25%) or best supportive care alone (16/56, 28%). Among the forty patients who received chemotherapy, the response rate to the first line was 22% (nine patients), while 30% (twelve patients) had stable disease, 35% (fourteen patients) had disease progression, and 12.5% (five patients) had missing data. The median overall survival for the whole cohort was 14 months (11.25–16.74). At last follow-up, 61 patients (83.6%) died of their disease, three (4%) died of unrelated causes, three (4%) were alive with disease, and five (6.8%) were alive without disease. Only one patient was lost to follow-up. The median follow-up was 85.7 months (16.8–158.4).

Full clinical characteristics according to the different SNPs are presented (S2 Data for APC I1307K, S3 Data for APC E1317Q, S4 Data for CD24 C170T, S5 Data for CD24 Tgdel, S6 Data for CD24 A1056G, S7 Data for CD24 A1626G).

### *APC* Incidence and Survival Data

**Fig 1:**
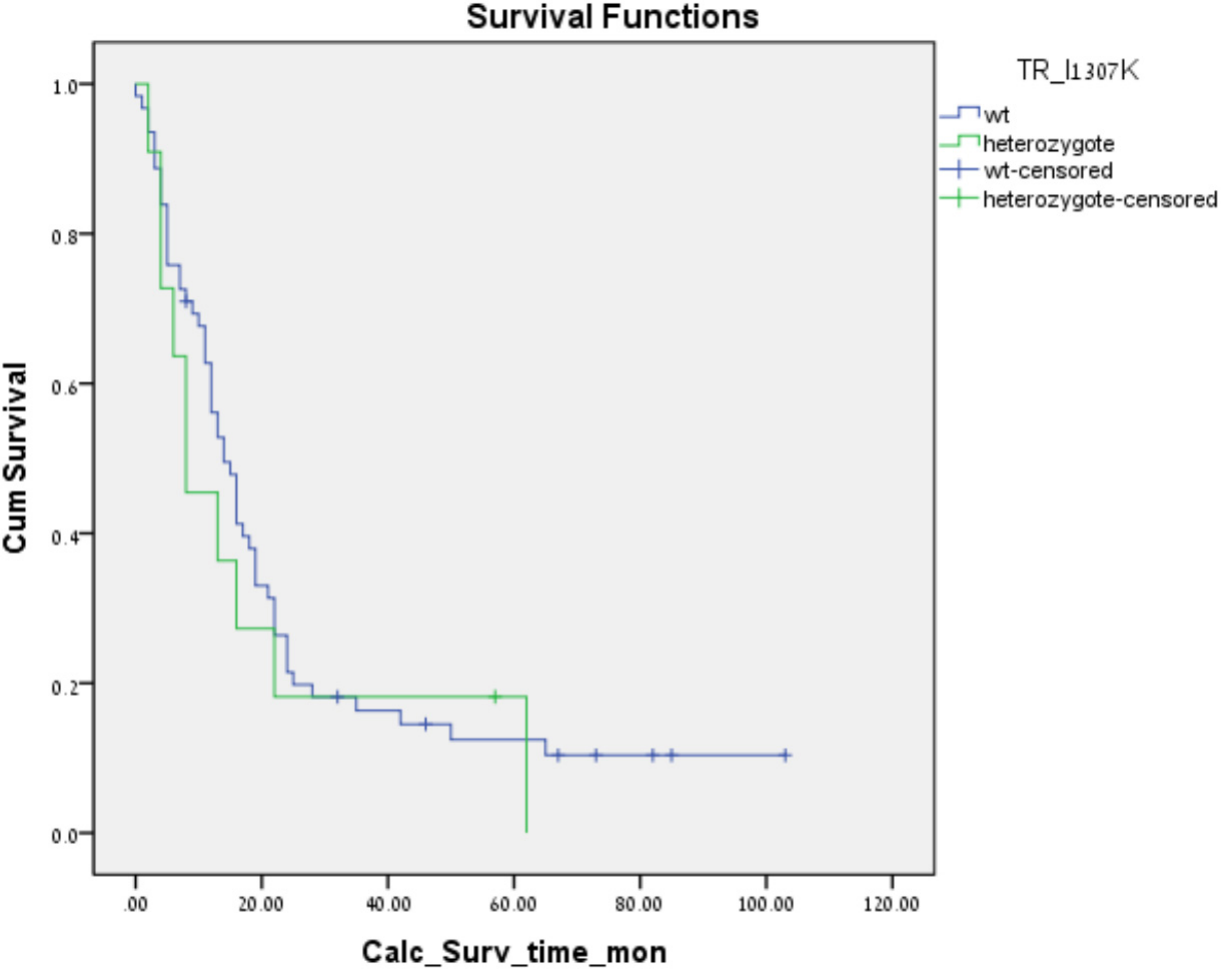
Kaplan-Meir survival curve for *APC* I1307K polymorphism.

The *APC* I1307K and E1317Q polymorphisms were identified in 11 (15%) and one (1.5%) patients, respectively. All the carriers in this cohort were heterozygotes of the *APC* polymorphisms. There was no statistically significant difference in survival between carriers of the various *APC* polymorphisms and non-carriers. The median survival of patients with WT *APC* I1307K was 14 months, as opposed to a median of eight months in the heterozygote group (Fig 1). However, the difference was not significant (*p* = 0.528). Only one patient was heterozygote for the *APC* E1317Q polymorphism, precluding the detection of a survival difference. Interestingly, that patient survived for much longer than the median, i.e., 28 months.

#### *CD24* incidence and survival data

*CD24* polymorphism 170^C→T^ was identified in 29 (39.7%) heterozygote patients and four (5.5%) homozygotes patients. The 1527^tgdel^ *CD24* variant was present in seven (9.6%) heterozygote patients and one (1.4%) homozygote patient. *CD24* polymorphism 1056^A→G^ was identified in 50 patients, 34 (46.6%) of whom were heterozygotes and 16 (21.9%) were homozygotes. The last *CD24* variant, 1626^A→G^, was present in 52 (71.2%) heterozygote patients and two (2.7%) homozygote patients (Table 2). There was no statistically significant difference in survival between carriers of the various *CD24* polymorphisms and non-carriers.

**Table 2:** Incidence of *APC* and *CD24* Polymorphisms between Patients.

	*APC* I1307K	*APC* E1317Q	*CD24* 170^C→T^	*CD24* 1527^tgde^	*CD24* 1056^A→G^	*CD24* 1626^A→G^
**Wild-type**	85%	96%	54.8%	87.7%	30.1%	24.7%
**Heterozygote**	15%	1.4%	39.7%	9.6%	46.6%	71.2%
**Homozygote**	-	-	5.5%	1.4%	21.9%	2.7%
**Missing**	-	2.6%	-	1.4%	1.4%	1.4%

**Fig 2:**
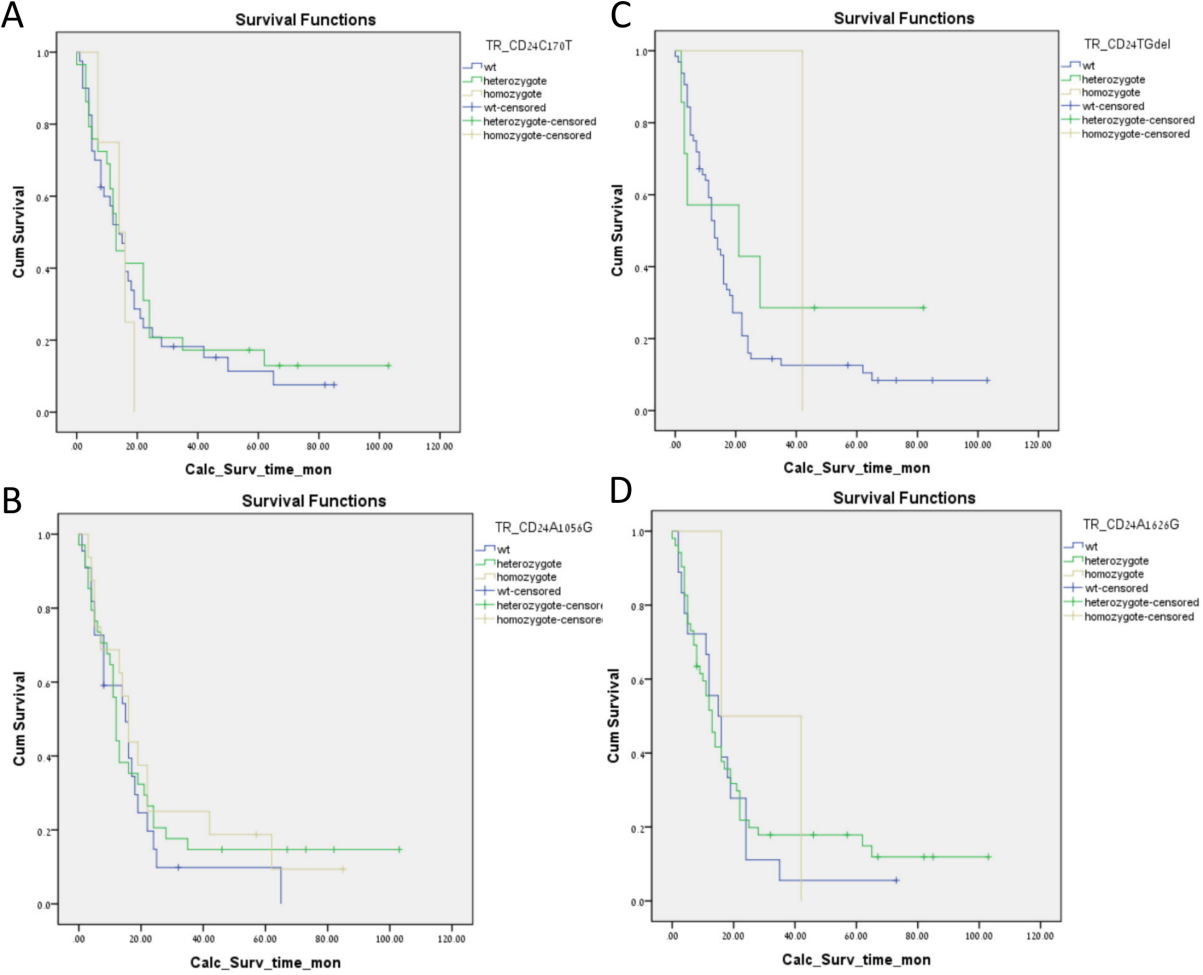
Kaplan-Meir survival curves. A. For *CD24* 170^C→T^ polymorphism. B For *CD24* 1527^tgdel^ polymorphism. C For *CD24* 1056^A→G^ polymorphism. D For *CD24* 1626^A→G^ polymorphism.

The median overall survival (mOS) of the *CD24* polymorphism 170^C→T^ heterozygotes and homozygotes was 13 and 14 months, respectively, compared to 14 months for the WT group (*p* = 0.722, Fig 2A). The mOS of the 1527^tgdel^ variant was 13 months for the WT group, 21 months for the heterozygote patients, and 42 months for the single homozygote patient (*p* = 0.494, Fig 2B). The 34 patients who were heterozygotes for *CD24* 1056^A→G^ polymorphism had a mOS of 12 months, as opposed to 15 months in the WT group and 16 months for the homozygote patients (*p* = 0.685, Fig 2C). The final group to be tested, those with the 1626^A→G^ variant, also failed to show any survival differences in comparison to the mOS of 15, 13 and 16 months in WT, heterozygote and homozygote patients, respectively (*p* = 0.834, Fig 2D).

### Genetic Permutations

**Fig 3:**
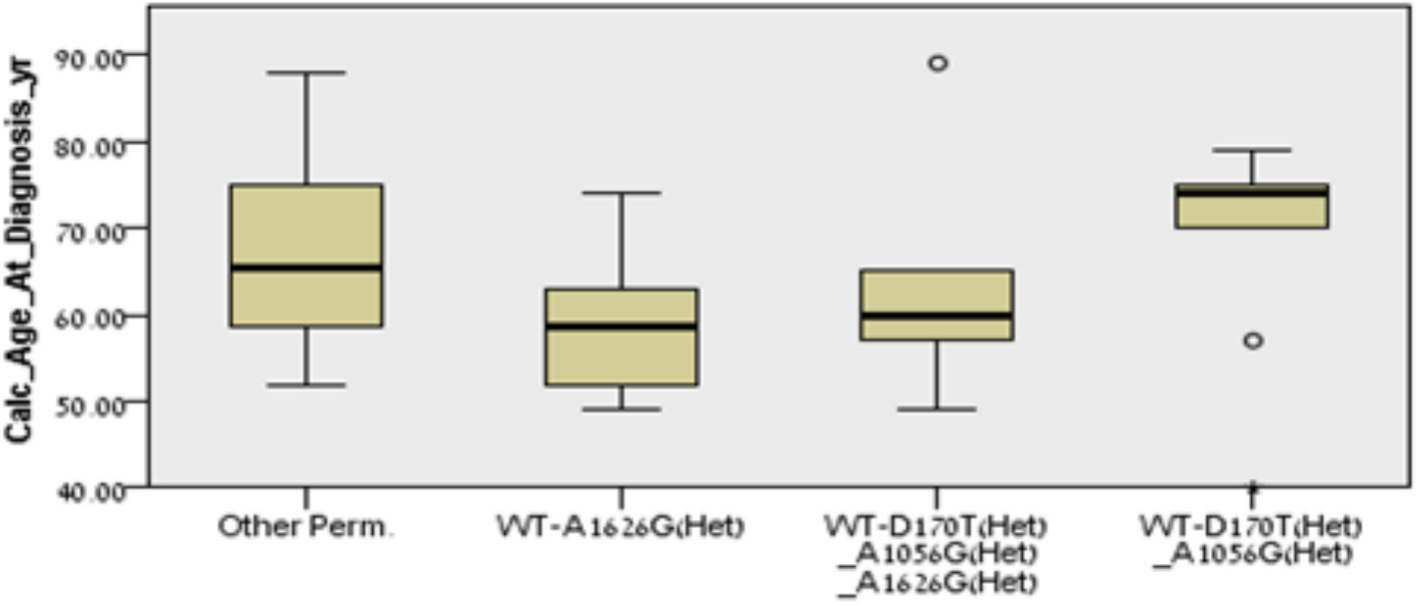
Independent samples Kruskal–Wallis test. Age differences between the patients with three predominant genotypes and the rest of the cohort.

We identified three genotypes which recurred in our cohort with high incidence. Genotype 1 (10/73 patients, 14% of the cohort) was WT to all six polymorphisms tested, with the exception of *CD24* 1626^A→G^ heterozygosity. Genotype 2 (10/73 patients, 14%) was WT to both *APC* variants and *CD24* 1527^tgdel^, and heterozygote to the remaining three *CD24* polymorphisms. The third group, genotype 3 (9/73 patients, 10%) was heterozygote to the *CD24* 170^C→T^ and 1056^A→G^ polymorphisms and WT to the rest. There was no statistical difference in survival between the patients with the three different genotypes and the rest of the cohort (*p* = 0.440). At the time of diagnosis, the median age of the 73 patients was 64 years, and there was no statistically significant difference between the various *APC* and *CD24* polymorphisms among them. However, when assessing the three dominant genotypes, the age at diagnosis was 58.5 years for genotype 1, 60 years for genotype 2, and 74 years for genotype 3, which was statistically significant compared to the rest of the cohort (*p* = 0.041, Fig 3). Comparison of additional clinical characteristics, such as response to first-line chemotherapy (*p* = 0.27), time until disease progression (*p* = 0.536) and tumor grade (*p* = 0.782) revealed that none was significantly different between the groups.

## Discussion

Ectopic expression of CD24 in tumor cells increases proliferation, promotes tumor cell adhesion to fibronectin, laminin and collagen I, IV (as well as P-selectin), and contributes to greater cell motility and invasiveness [5, 21]. Advances in recent research have linked CD24 to progression and malignancy of several epithelial and hematopoietic tumors. High expression of this protein was reported as being an independent predictor of poor survival in lung cancer [22–23]. Cytoplasmic staining for CD24 protein was absent in normal epithelium in ovarian cancer, and present in ovarian adenocarcinoma, and its overexpression was independently linked to a worse survival [24–25]. The same correlation was demonstrated in breast [26] and prostate cancers [27–28].

CD24 has an important oncogenic role in gastrointestinal tumors. Its overexpression was independently linked to poor prognosis in esophageal cancer [29–30]. Positive immunohistochemical staining for cytoplasmic CD24 was related to more advance staging in patients with gastric cancer [31]. A recent study showed that colon mucosa has an increased expression of CD24, even at an early stage of malignant transformation [21]. CD24 overexpression was correlated to decreased survival in colorectal cancer [32]. Sagiv et al. demonstrated a role of CD24 in the carcinogenetic process in pancreatic cancer [33]. In their study, human Colo357 pancreatic adenocarcinoma cells were treated *in vitro* with anti-CD24 mABs, which resulted in the arrest of cell growth in a dose- and time-dependent manner, while the cells negative to CD24 expression were not similarly affected. Anti-CD24 mABs were also effective in reducing tumor growth of bxpc3 pancreatic cancer xenografts in mice [34]. Pancreatic and colon adenocarcinoma cells treated *in vitro* with shRNA reportedly downregulated *CD24* expression and had impaired cell growth and motility [11]. The current study sought an association between four different *CD24* SNPs, two *APC* genetic variants, and the clinical course of pancreatic cancer. To the best of our knowledge, this is the first report on an investigation of these associations. There was no difference in the survival of heterozygous and homozygous patients, possibly due to the relatively small size of the cohort. Moreover, age at diagnosis, response to treatment, and time until disease progression also appeared to have no prognostic value in this setting.

The incidence of the four different *CD24* SNPs in our patient population was similar to its incidence among the healthy subjects described by our group in an earlier work [19], with the only significant exception having been found in the incidence of *CD24* 1626^A→G^: 29% of the healthy controls in that study were heterozygotes, compared to 71.2% of the PDA patients in the current work.

We had investigated the incidence of *APC* polymorphisms I1307K and E1317Q in healthy subjects in the past [20]. The incidence of E1317Q variant was 1.2% (twelve subjects out of 1000 screened), which is similar to our present result of 1.4% in PDA patients. The I1307K variant, however, was identified in only 5.3% healthy subjects, as compared with 15% in PDA patients.

Interestingly, out of many different genotype permutations, three stood out as being predominant in 14%, 14% and 10% of the patients. Two of the three genotypes, heterozygosity to *CD24* 1626^A→G^ (WT to the rest), and heterozygosity to *CD24* 170^C→T^, 1626^A→G^, 1527^tgdel^ (WT to the rest) were diagnosed at a significantly younger age than the rest of the group (median of 58.5 and 60 years compared with 64 years, respectively). The third genotype, the one with heterozygosity to *CD24* 170^C→T^ and 1056^A→G^ and WT to the rest, was diagnosed at a much older age (74 years).

## Conclusion

Although we were unable to detect any impact of *APC* and *CD24* genes on survival in patients with PDA, the data shown here warrant further study with regard to their role in pancreatic carcinogenesis and prognosis, and in a larger cohort.

## Supporting Information

S1 Data Statistical calculations, along with actual sample sizes per SNP. (TIF)

S2 Data Tumor stage, grade and location for APC I1307K polymorphism. (TIF)

S3 Data Tumor stage, grade and location for APC E1317Q polymorphism. (TIF)

S4 Data Tumor stage, grade and location for CD24 C170T polymorphism. (TIF)

S5 Data Tumor stage, grade and location for CD24 Tgdel polymorphism. (TIF)

S6 Data Tumor stage, grade and location for CD24 A1056G polymorphism. (TIF)

S7 Data Tumor stage, grade and location for CD24 A1626G polymorphism. (TIF)
