# Supplementary figures and images for: CD24 and APC Genetic Polymorphisms in Pancreatic Cancers as Potential Biomarkers for Clinical Outcome
Source: PLoS One. 2015 Sep 22;10(9):e0134469. doi: 10.1371/journal.pone.0134469 (PMC4579075; doi:10.1371/journal.pone.0134469)

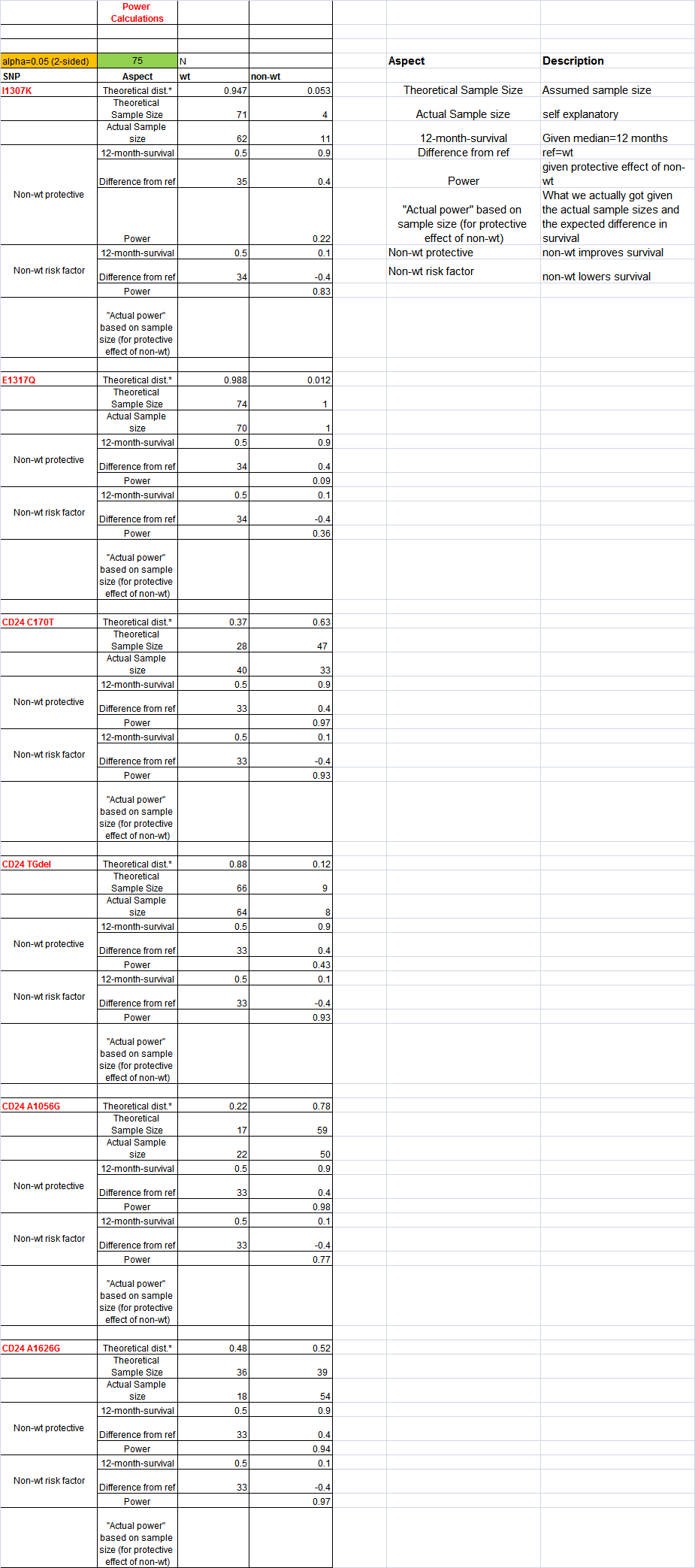

Supplement: S1 Data — (TIF) [file pone.0134469.s001.TIF]

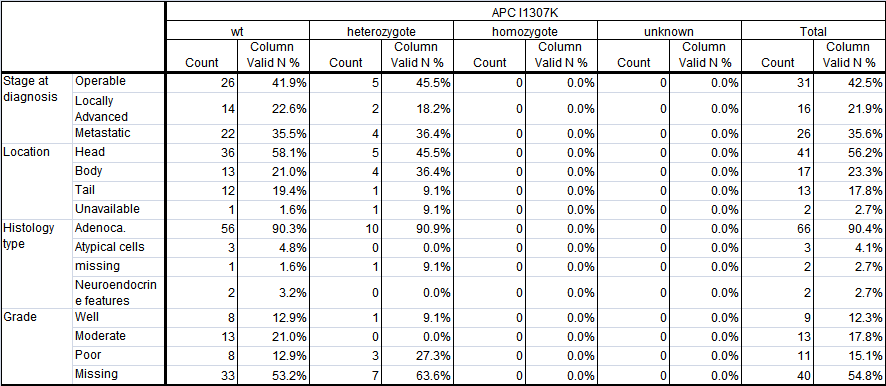

Supplement: S2 Data — (TIF) [file pone.0134469.s002.TIF]

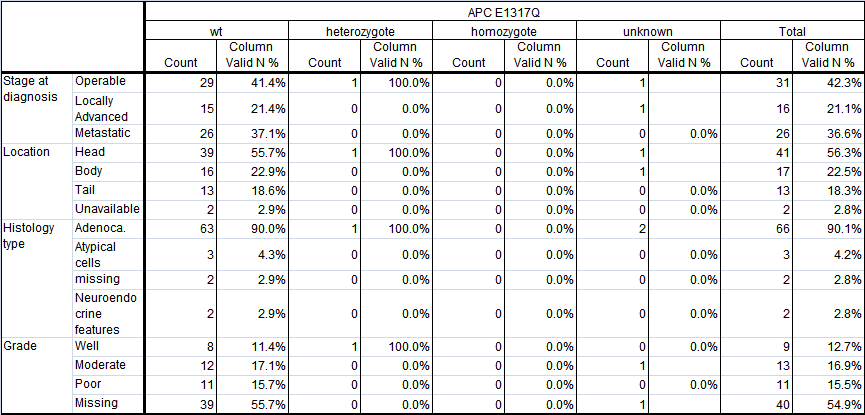

Supplement: S3 Data — (TIF) [file pone.0134469.s003.TIF]

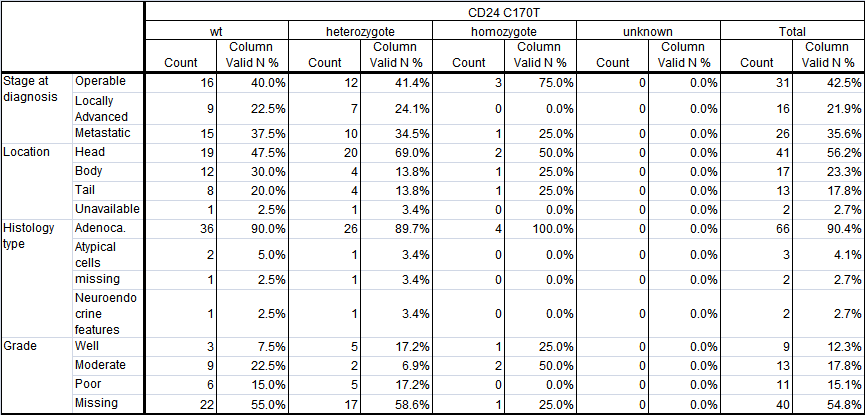

Supplement: S4 Data — (TIF) [file pone.0134469.s004.TIF]

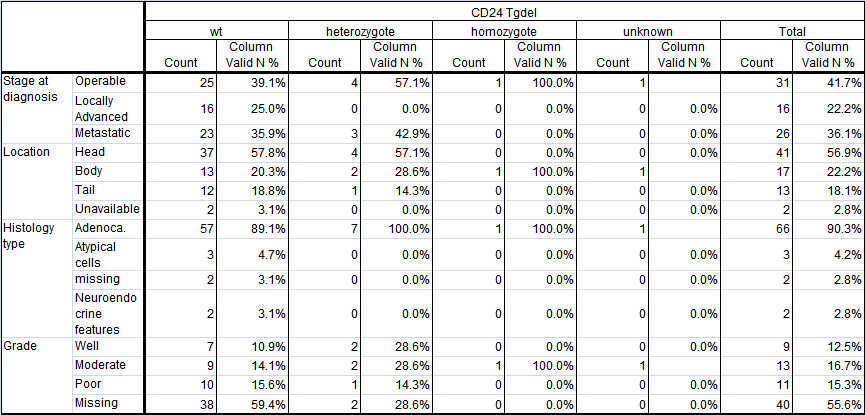

Supplement: S5 Data — (TIF) [file pone.0134469.s005.TIF]

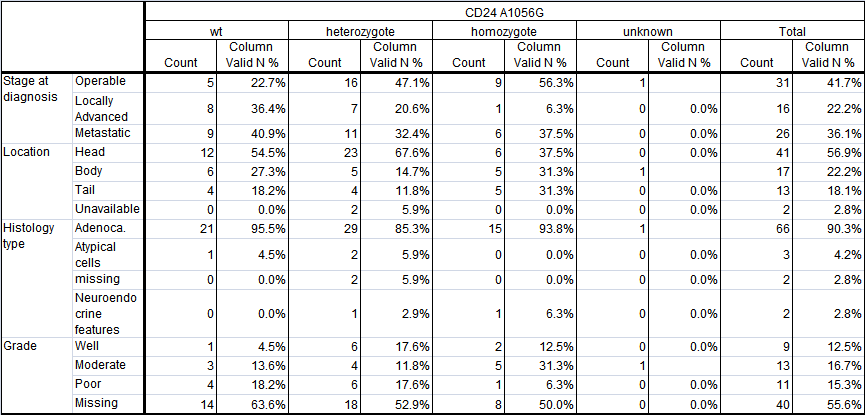

Supplement: S6 Data — (TIF) [file pone.0134469.s006.TIF]

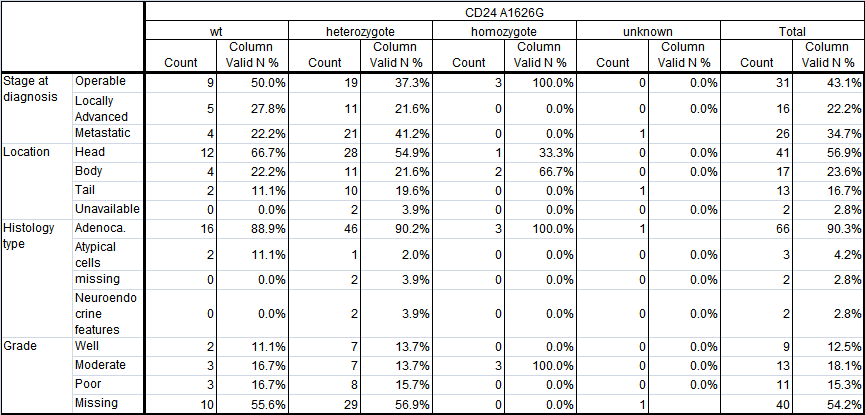

Supplement: S7 Data — (TIF) [file pone.0134469.s007.TIF]
